# Prognostic value of the recovery time of continuous normal voltage in amplitude-integrated electroencephalography in out-of-hospital cardiac arrest patients treated with therapeutic hypothermia: a retrospective study

**DOI:** 10.1186/s40560-016-0152-5

**Published:** 2016-04-02

**Authors:** Kazuhiro Sugiyama, Masahiro Kashiura, Akiko Akashi, Takahiro Tanabe, Yuichi Hamabe

**Affiliations:** Trauma and Critical Care Center, Tokyo Metropolitan Bokutoh Hospital, 23-15 Kotobashi, 4-Chome, Sumida-ku, Tokyo, 130-8575 Japan

**Keywords:** Amplitude-integrated electroencephalography, Cardiac arrest, Neurological outcome, Therapeutic hypothermia

## Abstract

**Background:**

The early prediction of neurological outcomes in postcardiac arrest patients treated with therapeutic hypothermia (TH) remains challenging. Amplitude-integrated electroencephalography (aEEG) is a type of quantitative EEG. A particular cutoff time from the return of spontaneous circulation (ROSC) to the recovery of a normal aEEG trace for predicting a good neurological outcome has not yet been established. The purpose of the present study was to examine the relation between neurological outcomes and the continuous normal voltage (CNV) recovery time in adult comatose survivors of cardiac arrest treated with TH and identify the recovery time cutoff for predicting a good neurological outcome.

**Methods:**

We retrospectively evaluated adult survivors of cardiac arrest with initial shockable rhythm treated with TH and monitored with aEEG. A good outcome was defined as a cerebral performance category (CPC) of 1 or 2 at hospital discharge. A CNV trace was considered as the normal aEEG trace, and the CNV recovery time was defined as the time from ROSC to the initial CNV trace.

**Results:**

The study included 30 patients, and of these patients, 22 had recovery of CNV trace. The median CNV recovery time was shorter among patients with a good outcome than that among those with a poor outcome (10.7 h [interquartile range (IQR), 7.4–15.8 h] vs. 28.6 h [IQR, 26.9–29.3 h]; *p* = 0.003). The area under the receiver operating characteristic curve of the CNV recovery time for predicting a good neurological outcome was 0.95 (95 % CI 0.86–1; *p* = 0.003), and the optimal cutoff was 23 h. The recovery of CNV trace within 23 h had a sensitivity of 89 %, specificity of 100 %, positive predictive value of 100 %, and negative predictive value of 86 % for predicting a good neurological outcome in all the patients, including the eight patients without recovery of CNV trace.

**Conclusions:**

A CNV recovery time cutoff of 23 h might help predict a good neurological outcome in adult survivors of cardiac arrest treated with TH.

## Background

The early prediction of neurological outcomes in postcardiac arrest patients treated with therapeutic hypothermia (TH) remains challenging. Recently, continuous electroencephalography (cEEG) was reported to be useful in the early prediction of neurological outcomes [1]. However, special training is required for the interpretation of cEEG findings. Amplitude-integrated electroencephalography (aEEG) is a type of quantitative EEG and is derived from single- or two-channel cEEG recordings. The maximum and minimum amplitudes within a short period are displayed as bandwidths along with a compressed time scale. aEEG findings can be easily interpreted by non-neurologists, and aEEG findings have already been widely used to predict neurological outcomes in neonates with hypoxic-ischemic encephalopathy [2]. Recently, Rundgren et al. [3] and Oh et al. [4] reported the usefulness of aEEG for predicting neurological outcomes in adult comatose survivors of cardiac arrest treated with TH. The recording channels and pattern classifications of aEEG differed between these two reports. Rundgren et al. classified aEEG patterns as follows: continuous, flat trace (FT), burst suppression (BS), and electrical status epilepticus (SE), while Oh et al. classified aEEG patterns as follows: continuous normal voltage (CNV), discontinuous normal voltage (DNV), low voltage (LV), FT, BS, and SE. The classification used by Oh et al. is commonly applied in the field of neonatology and has a more precise voltage criterion than the classification used by Rundgren et al. The continuous pattern in the classification used by Rundgren et al. and the CNV pattern used by Oh et al. were found to provide the best trace in each classification, which could be considered as a normal trace for cardiac arrest survivors. Both these studies showed that the early recovery of this normal aEEG trace after cardiac arrest predicts a good neurological outcome. However, a BS pattern and the lack of recovery of the normal trace within 36–72 h after cardiac arrest predicted a poor neurological outcome. An initial FT pattern of aEEG does not necessarily predict a poor outcome if the normal trace recovers during this initial period.

Although recovery of the normal aEEG trace is essential to obtain a good neurological outcome, some patients who take a long time to achieve recovery of the normal trace do not have a good neurological outcome. A particular cutoff time from the return of spontaneous circulation (ROSC) to the recovery of the normal aEEG trace for predicting a good neurological outcome has not yet been established. The purpose of the present study was to examine the relation between neurological outcomes and the CNV recovery time in adult comatose survivors of cardiac arrest treated with TH, and identify the recovery time cutoff for predicting a good neurological outcome.

## Methods

### Patients

This retrospective study included comatose survivors of out-of-hospital cardiac arrest aged 18 years or older, who were admitted to the emergency department of Tokyo Metropolitan Bokutoh Hospital in Japan and were treated with TH after successful resuscitation between January 2013 and December 2014. Patients with initial non-shockable rhythm were excluded from the study. Because the present study used aEEG recordings in the early phase after cardiac arrest, patients in whom aEEG monitoring was started ≥24 h after ROSC were excluded. Additionally, patients with a history of neurological diseases or brain injury, and those who died within 72 h after cardiac arrest were excluded. The baseline demographic and clinical characteristics of the patients were collected from medical records, and the timings of prehospital events were recorded according to the reports of emergency medical service personnel. The institutional review board of Tokyo Metropolitan Bokutoh Hospital approved the study, and the requirement of informed consent was waived owing to the retrospective design of the study.

### TH protocol

All patients were resuscitated according to the current recommendations.

Patients who remained comatose (lack of meaningful responses to verbal commands) after ROSC were treated with TH at 34 °C for 24 h. Exclusion criteria of TH included hemodynamic instability refractory to the use of vasopressor agents and mechanical support, refractory ventricular arrhythmia (ventricular fibrillation and sustained ventricular tachycardia), active bleeding, and terminal illness prior to cardiac arrest. TH was started immediately after admission to the emergency department and was applied by using a cooling technique combining the administration of intravenous ice-cold fluids and the application of a surface cooling device (Arctic Sun System; Medivance, Louisville, CO). Extracorporeal cardiopulmonary resuscitation (ECPR) was performed in witnessed cardiac arrest patients with initial shockable rhythm, who were aged ≤65 years, if cardiac arrest sustained and was refractory to conventional advanced life support procedures after hospital arrival. Exclusion criteria of ECPR included the prolonged cardiac arrest ≥30 min before hospital arrival and terminal illness prior to cardiac arrest. In these patients, a heat exchanger was used in the circuit to control body temperature. Bladder temperature was used for temperature management. Midazolam (0.05–0.1 mg/kg/h) and fentanyl (1 μg/kg/h) were administered for sedation and analgesia, and vecuronium (0.05–0.1 mg/kg/h) was administered to control shivering. After completion of 24 h of TH, patients were rewarmed at the rate of 0.15 °C/h until the body temperature reached 36 °C, and then, midazolam and vecuronium were discontinued.

### aEEG data

**Fig. 1:**
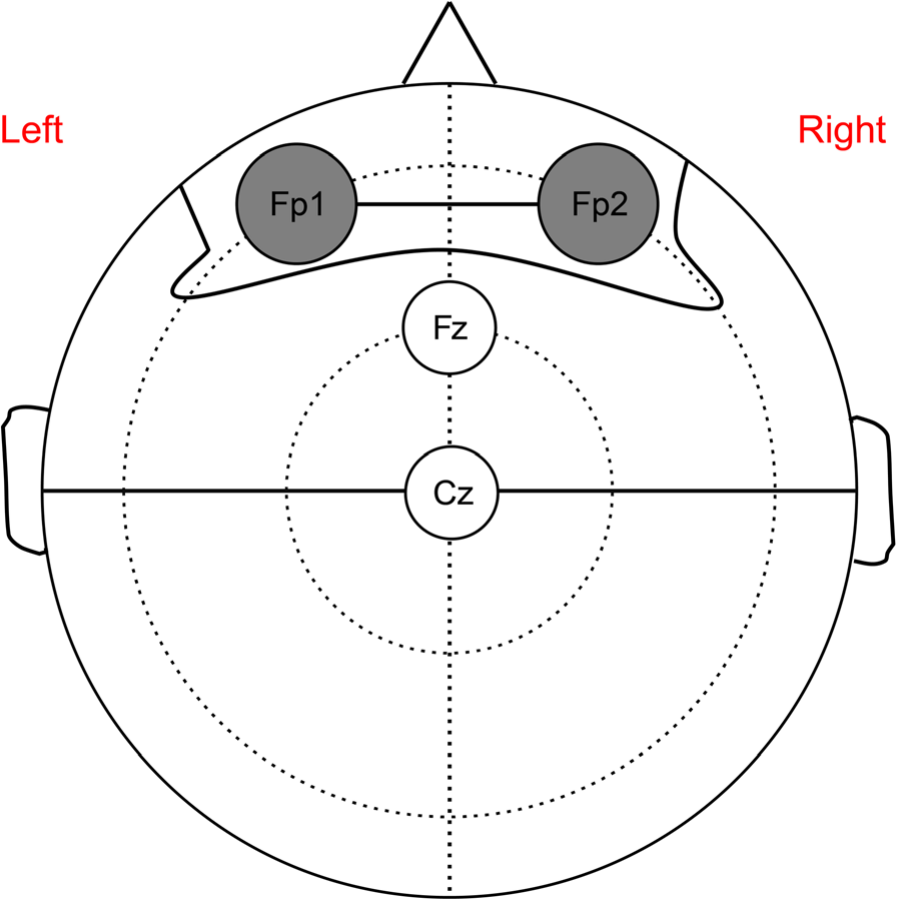
Placement of electrodes for amplitude-integrated electroencephalography (aEEG) monitoring. Cup electrodes are attached at positions *Fp1* and *Fp2*, and aEEG monitoring was performed for the bipolar channel Fp1-Fp2

All patients were monitored with aEEG (NicoletOne; Viasys Healthcare, Madison, WI) after admission to the intensive care unit. The cup electrodes were attached across the forehead at positions Fp1 and Fp2, and aEEG monitoring was performed for the bipolar channel Fp1-Fp2 (Fig. 1).

**Fig. 2:**
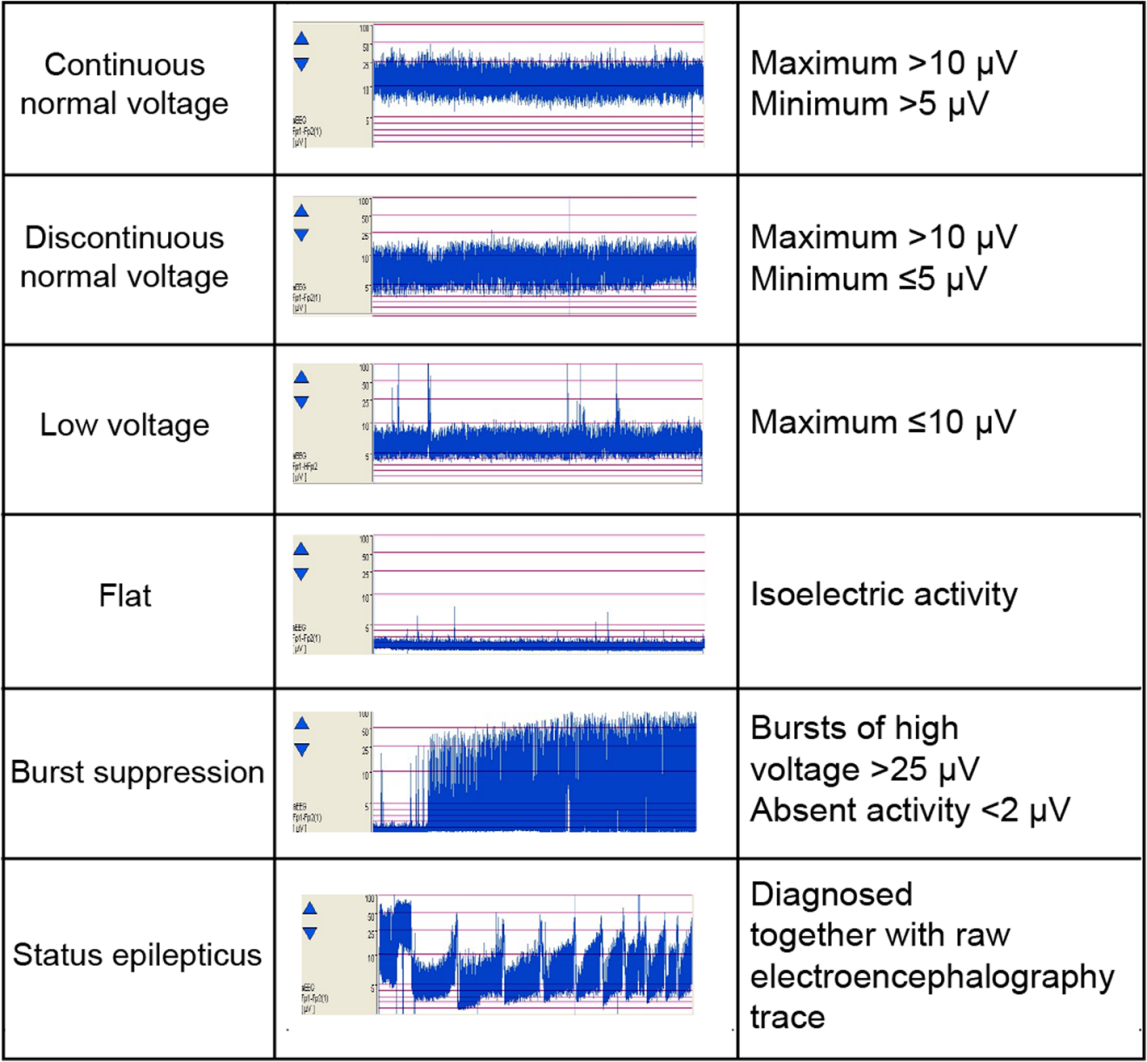
Classification system of the patterns of amplitude-integrated electroencephalography traces used in the study

We applied the classification system of aEEG patterns used by Oh et al. (Fig. 2) [4], and a CNV trace was considered the normal trace in both pattern and voltage criteria.

The CNV recovery time was defined as the time from ROSC to the initial recoding of a CNV trace (maximum amplitude >10 μV; minimum amplitude >5 μV) on aEEG with a time scale of 6 cm/h. The CNV recovery time was examined in all patients with a CNV trace on aEEG. In patients with a CNV trace at the start of aEEG monitoring, the CNV recovery time was defined as the time from ROSC to the start of aEEG monitoring.

### Neurological outcome assessment

The cerebral performance category (CPC) scale was used to assess neurological outcomes on the day of hospital discharge, and the data were extracted from the medical records. The neurological outcome was considered good if the CPC was 1 or 2, and poor if the CPC was 3–5 [5].

### Statistical analysis

Continuous variables are reported as medians with interquartile ranges (IQRs), and dichotomous variables are reported as numbers with percentages. Univariate comparisons of the outcomes were performed using the Fisher exact test for categorical variables and the Mann-Whitney *U* test for continuous variables. The optimal CNV recovery time cutoff for predicting a good neurological outcome was determined using the Youden index. The ability to predict the neurological outcome was assessed according to sensitivity, specificity, positive predictive value (PPV), negative predictive value (NPV), and area under the receiver operating characteristic curve (AUROC). All statistical analyses were performed using EZR (Saitama Medical Center, Jichi Medical University, Saitama, Japan), which is a graphical user interface for R (The R Foundation for Statistical Computing, Vienna, Austria) [6].

## Results

**Fig. 3:**
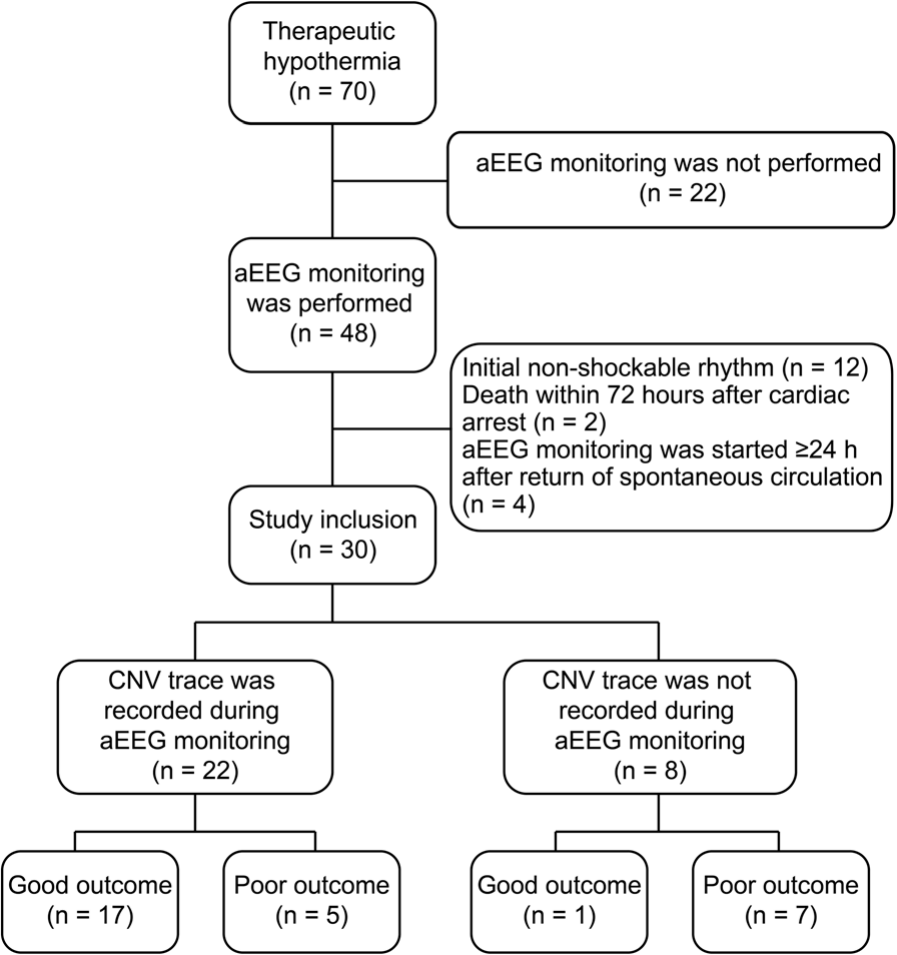
Flow chart of patient selection. *aEEG* amplitude-integrated electroencephalography, *CNV* continuous normal voltage

During the study period, 70 out-of-hospital cardiac arrest patients were treated with TH. Of these patients, 48 were monitored with aEEG. However, 12 with initial non-shockable rhythm, two who died within 72 h after cardiac arrest, and four without aEEG monitoring in the first 24 h after ROSC were excluded. Therefore, 30 patients were finally included in the study (Fig. 3). Of the 30 patients, 27 (90 %) were male. The median age of the patients was 62 years (IQR, 45–71 years), and 26 (87 %) patients had a witnessed collapse. The median time from collapse to ROSC was 22 min (IQR, 18–40 min). The median time from collapse to the start of aEEG monitoring was 6.8 h (IQR, 4.8–8.5 h), and the median duration of aEEG monitoring was 50 h (IQR, 45–63 h). A total of four (13 %) patients received ECPR. Of the 30 patients, 18 (60 %) had a good outcome and 12 (40 %) had a poor outcome. The median age was significantly lower among patients with a good outcome than among those with a poor outcome (47 years [IQR, 43–66 years] vs. 70 years [IQR, 62–72 years]; *p* = 0.01). Additionally, the median time from collapse to ROSC was significantly shorter among patients with a good outcome than among those with a poor outcome (19 min [IQR, 14–22 min] vs. 44 min [IQR, 30–47 min]; *p* < 0.001). Moreover, the proportion of patients who received ECPR was significantly lower among patients with a good outcome than among those with a poor outcome (0 vs. 33 %; *p* = 0.02). Furthermore, the median time from collapse to the target temperature was significantly longer among patients with a good outcome than among those with a poor outcome (6.1 h [IQR, 5.3–8.0 h] vs. 4.0 h [IQR, 1.3–5.7 h]; *p* = 0.02). Other variables were comparable between patients with a good outcome and those with a poor outcome (Table 1). At discharge, 18 (60 %) patients had a CPC of 1 or 2, six (20 %) had a CPC of 3 or 4, and six (20 %) had a CPC of 5 (died in hospital). The median duration of hospital stay was 24 days (IQR, 12–41 days) among all the patients, 22 days (IQR, 13–34 days) among those with a good outcome, and 42 days (IQR, 5–56 days) among those with a poor outcome.

**Table 1:** Comparison of patient characteristics between patients with good and poor neurological outcomes

	All patients (*n* = 30)	Good outcome (*n* = 18)	Poor outcome (*n* = 12)	*p* value
Age (years)^a^	62 (45–71)	47 (43–66)	70 (62–72)	0.01^b^
Male, number (%)	27 (90 %)	15 (83 %)	12 (100 %)	0.26^c^
Witnessed collapse	26 (87 %)	16 (89 %)	10 (83 %)	1^c^
Time from collapse to				
ROSC (min)^a^	22 (18–40)	19 (14–22)	44 (30–47)	<0.001^b^
Target temperature (h)^a^	5.5 (4.2–6.7)	6.1 (5.3–8.0)	4.0 (1.3–5.7)	0.02^b^
aEEG monitoring (h)^a^	6.8 (4.8–8.5)	6.1 (4.7–8.2)	6.9 (5.3–9.3)	0.35^b^
Duration of aEEG monitoring (h)^a^	50 (45–63)	53 (46–58)	47 (45–68)	0.90^b^
Coronary angiography, number (%)	28 (93 %)	17 (94 %)	11 (92 %)	1^c^
ECPR, number (%)	4 (13 %)	0 (0 %)	4 (33 %)	0.02^c^

*ROSC* return of spontaneous circulation, *aEEG* amplitude-integrated electroencephalography, *CPC* cerebral performance category, *ECPR* extracorporeal cardiopulmonary resuscitation

^a^Median (interquartile range)

^b^Mann-Whitney *U* test

^c^Fisher’s exact test

**Fig. 4:**
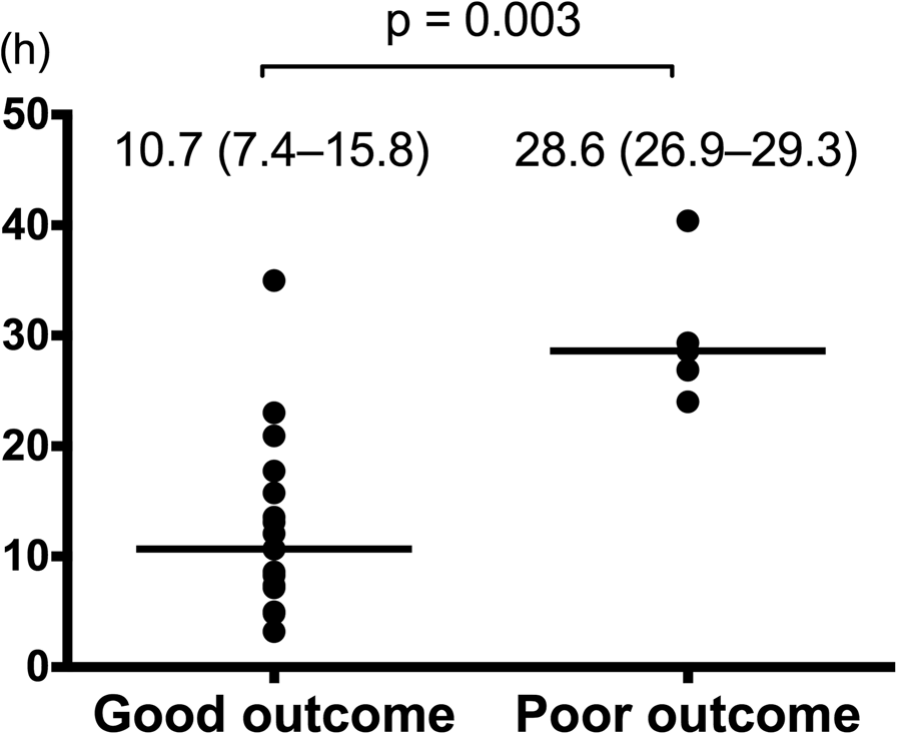
Recovery time of CNV between patients with good outcomes and those with poor outcomes. The CNV recovery time is presented as median (interquartile range). The Mann-Whitney *U* test was used for the comparison. *CNV* continuous normal voltage

**Fig. 5:**
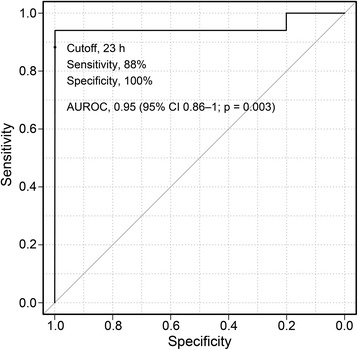
ROC curve of the CNV recovery time to predict a good neurological outcome. *ROC* receiver operating characteristic, *CNV* continuous normal voltage, *AUROC* area under the receiver operating characteristic curve, *CI* confidence interval

As the initial trace, eight patients had CNV trace, three had DNV, seven had LV, one had BS, one had SE, and 10 had FT. All the eight patients of initial CNV trace, two of DNV, five of LV, and three of FT had a good neurological outcome. During aEEG monitoring, 22 patients had recovery of CNV trace, and of these patients, 17 (77 %) had a good neurological outcome. Additionally, of the 22 patients, eight had CNV trace as the initial trace, two had DNV, five had LV, one had SE, and six had FT. In this group of patients who had recovery of CNV trace, the median CNV recovery time was shorter among patients with a good outcome than among those with a poor outcome (10.7 h [IQR, 7.4–15.8 h] vs. 28.6 h [IQR, 26.9–29.3 h]; *p* = 0.003) (Fig. 4). The AUROC of the CNV recovery time for predicting a good neurological outcome was 0.95 (95 % CI 0.86–1; *p* = 0.003), and the optimal recovery time cutoff was 23 h (Fig. 5).

In all the 30 patients, including the eight patients without recovery of CNV trace, the recovery of CNV trace within 23 h could predict a good neurological outcome, with a sensitivity of 89 %, specificity of 100 %, PPV of 100 %, and NPV of 86 % (Table 2).

**Table 2:** Sensitivity, specificity, PPV, and NPV of the CNV recovery time cutoff of 23 h

	Good outcome (*n*)	Poor outcome (*n*)	Sensitivity (95 % CI)	Specificity (95 % CI)	PPV (95 % CI)	NPV (95 % CI)
CNV recovery time ≤23 h	16	0	89 (65–97)	100 (64–100)	100 (71–100)	86 (57–98)
CNV recovery time >23 h or no CNV trace	2	12				

*CNV* continuous normal voltage, *PPV* positive predictive value, *NPV* negative predictive value, *CI* confidence interval

## Discussion

The present study found that the CNV recovery time is useful for predicting neurological outcomes in comatose survivors of cardiac arrest treated with TH and that the optimal CNV recovery time cutoff for predicting a good neurological outcome was 23 h.

After severe brain ischemia, EEG readings are initially suppressed and recover gradually. Thoresen et al. [7] reported that the time to a normal aEEG trace was a good predictor of neurological outcomes in infants with asphyxia. Our study showed that this is also true in adult postcardiac arrest patients.

Several factors, such as hypothermia, sedation, and the method of aEEG recording, should be considered in the evaluation of the CNV recovery time. EEG has been reported to not be significantly affected at a body temperature of 32–34 °C [8]. Midazolam is a sedative that is widely used during TH, and it has been reported to cause an increase in beta activity on EEG [9]. Additionally, midazolam can decrease the voltage of aEEG recordings in neonates [10]. Moreover, in critically ill adult patients, midazolam can attenuate the voltage of EEG [11]. In our study, midazolam was infused according to body weight; therefore, the effect of midazolam on the amplitude of EEG would be relatively uniform in all patients. However, if high doses of midazolam or other sedatives, such as propofol and barbiturates, are administered during TH, the CNV recovery time cutoff for predicting a good neurological outcome may differ from that identified in the present study. Additionally, in older patients or those with multiple organ dysfunctions, the CNV recovery time may be prolonged owing to delayed metabolism or excretion of drugs, or the effect of metabolic encephalopathy. Therefore, a long CNV recovery time does not necessarily exclude the possibility of a good neurological outcome. In our study, a patient with a CNV recovery time of 35 h had a good neurological outcome. On the other hand, few factors have been reported to shorten the CNV recovery time. Therefore, a short CNV recovery time is strongly associated with a good neurological outcome, with a high specificity and PPV. We believe that the CNV recovery time should be used as a predictor of good neurological outcome in comatose survivors of cardiac arrest. However, the possibility of alpha coma should be considered when we evaluate patients with short CNV recovery time. Alpha coma without reactivity to stimuli has been reported to be associated with a poor neurological outcome [12], and the aEEG trace may be classified as a CNV pattern in patients experiencing alpha coma. Although no patient had alpha coma in the present study and the precise frequency of alpha coma in postcardiac patients is not known, alpha coma can lower the specificity and PPV of the CNV recovery time to predict a good neurological outcome.

The method of aEEG has not been standardized in adult postcardiac arrest patients. Needle electrodes are not popular in Japan; therefore, we used cup electrodes in the present study. The cup electrodes were placed across the forehead, and a bipolar frontal channel was used for aEEG monitoring. In the study by Rundgren et al. [3], the bipolar channels F3-P3 and F4-P4 were used; however, frontal channels are easier to place, less likely to detach from the forehead, and more suitable for use in postcardiac arrest patients when cup electrodes are used. Physicians can attach these cup electrodes easily within a short period. Focal seizures may not be recorded with frontal channels alone; however, the monitoring of global cerebral activity is possible with frontal channels alone in postcardiac arrest patients [4].

The classifications used by Rundgren et al. [3] and Oh et al. [4] differed. However, both classifications can be easily applied by non-neurologists. The classification system used by Oh et al. has a more precise voltage criteria and it adopts CNV, DNV, and LV to further classify the continuous pattern in the classification system used by Rundgren et al. In the study by Oh et al., the specificity and PPV of the initial aEEG trace to predict a good neurological outcome were higher with the voltage method than with the pattern method alone. Therefore, we applied the classification system used by Oh et al. and considered a CNV trace as the normal trace in order to obtain a high specificity and PPV.

In comatose survivors of cardiac arrest treated with TH, some reliable predictors of a poor neurological outcome have been reported [13], which help physicians avoid providing futile treatment. However, predictors of a good neurological outcome with a high specificity are scarce. If a good neurological outcome can be predicted at the early phase after cardiac arrest, physicians can select patients having the potential of neurological recovery for invasive and advanced treatments that have a high cost. Therefore, the use of the CNV recovery time cutoff can help in the appropriate treatment of postcardiac arrest patients.

### Limitations

The present study has several limitations. First, this was a retrospective study performed at a single center, with a small sample size. The timing of the start of aEEG monitoring and the duration of monitoring were not determined previously. Several patients had a CNV trace at the start of monitoring; therefore, the actual median CNV recovery time of patients with a good outcome may have been overestimated. However, this does not affect the CNV recovery time cutoff. Second, physicians were not blinded to the aEEG results during treatment. Although the aEEG results were not used to determine withdrawal of therapy, the aEEG results might have influenced therapy selection. Third, the CPC was determined at discharge, and recovery after discharge was not considered. Additionally, the patients who died within 72 h after cardiac arrest were excluded because neurological outcomes could not be conclusively assessed in these patients. Six patients died in the hospital in the present study, and they were included in the poor outcome group. Among these six patients, five showed diffuse brain swelling or loss of white and gray matter on head CT and one remained comatose without motor response to stimuli and died from sudden circulatory collapse on day 5 (3 days after normothermia). Fourth, the present study only included patients with initial shockable rhythm in order to unify the sample conditions. Survivors of cardiac arrest with initial non-shockable rhythm were excluded as prognoses have been reported to differ between patients with initial shockable and those with non-shockable rhythm [14, 15]. These patients should be evaluated separately in future studies.

## Conclusions

Neurological outcomes might be related with the CNV recovery time, and a CNV recovery time cutoff of 23 h might help predict a good neurological outcome in comatose survivors of cardiac arrest with initial shockable rhythm who are treated with TH.
